# Correction: Conformationally restricted benzothienoazepine respiratory syncytial virus inhibitors: their synthesis, structural analysis and biological activities

**DOI:** 10.1039/c8md90016g

**Published:** 2018-03-26

**Authors:** Euan A. F. Fordyce, S. Fraser Hunt, Damien Crepin, Stuart T. Onions, Guillaume F. Parra, Chris J. Sleigh, John King-Underwood, Harry Finch, John Murray

**Affiliations:** a Sygnature Discovery Ltd. , BioCity , Nottingham , NG1 1GF , UK . Email: e.fordyce@sygnaturediscovery.com ; Tel: +44 (0)1159415401; b CompChem Resource , Old Cottage Hospital , Homend , Ledbury , Herefordshire , HR8 1ED , UK; c Pulmocide Ltd. , 52 Princes Gate, Exhibition Road, South Kensington , London , SW7 2PG , UK . Email: john@pulmocide.com ; Tel: +44 (0)2037639484

## Abstract

Correction for ‘Conformationally restricted benzothienoazepine respiratory syncytial virus inhibitors: their synthesis, structural analysis and biological activities’ by Euan A. F. Fordyce *et al.*, *Med. Chem. Commun.*, 2018, DOI: ; 10.1039/c8md00033f.



## 

The authors regret that the information in note 17 in the References section of the manuscript gives an incorrect ratio of the isomers. The correct information is as follows:

17. The major impurity present in the mixture (29% isolated yield) was the corresponding propenyl thiophene **24**, present as a 4 : 1 mixture of *trans* : *cis* isomers, which presumably form *via* elimination of triphenylphosphine oxide from the intermediate alkyloxytriphenylphosphonium species.

In addition, the wording in the Acknowledgements should be amended as follows:

The authors would like to thank Dr Kaz Ito (Pulmocide Ltd.) and Heather Allen (Pulmocide Ltd.) for the provision of the biological screening data for compounds **8a**, **14** and **20**.

The Royal Society of Chemistry apologises for these errors and any consequent inconvenience to authors and readers.

